# Role of N-cadherin in proliferation, migration, and invasion of germ cell tumours

**DOI:** 10.18632/oncotarget.5288

**Published:** 2015-10-02

**Authors:** Felix Bremmer, Simon Schallenberg, Hubertus Jarry, Stefan Küffer, Silke Kaulfuss, Peter Burfeind, Arne Strauβ, Paul Thelen, Heinz Joachim Radzun, Philipp Ströbel, Friedemann Honecker, Carl Ludwig Behnes

**Affiliations:** ^1^ Institute of Pathology, University of Göttingen, Göttingen, Germany; ^2^ Department of Endocrinology, University Medical Center Göttingen, Göttingen, Germany; ^3^ Department of Human Genetics, University of Göttingen, Göttingen, Germany; ^4^ Department of Urology, University of Göttingen, Göttingen, Germany; ^5^ Department of Oncology, University Medical Center Hamburg-Eppendorf, University of Hamburg, Hamburg, Germany; ^6^ Tumour and Breast Center ZeTuP, St. Gallen, Switzerland

**Keywords:** N-cadherin, cisplatin resistance, germ cell tumours, GCT-cell lines

## Abstract

Germ cell tumors (GCTs) are the most common malignancies in young men. Most patients with GCT can be cured with cisplatin-based combination chemotherapy, even in metastatic disease. In case of therapy resistance, prognosis is usually poor. We investigated the potential of N-cadherin inhibition as a therapeutic strategy. We analyzed the GCT cell lines NCCIT, NTERA-2, TCam-2, and the cisplatin-resistant sublines NCCIT-R and NTERA-2R. Effects of a blocking antibody or siRNA against N-cadherin on proliferation, migration, and invasion were investigated. Mouse xenografts of GCT cell lines were analyzed by immunohistochemistry for N-cadherin expression. All investigated GCT cell lines were found to express N-cadherin protein *in vitro* and *in vivo*. Downregulation of N-cadherin *in vitro* leads to a significant inhibition of proliferation, migration, and invasion. N-cadherin-downregulation leads to a significantly higher level of pERK. N-cadherin-inhibition resulted in significantly higher rates of apoptotic cells in caspase-3 staining. Expression of N-cadherin is preserved in cisplatin-resistant GCT cells, pointing to an important physiological role in cell survival. N-cadherin-downregulation results in a significant decrease of proliferation, migration, and invasion and stimulates apoptosis in cisplatin-naive and resistant GCT cell lines. Therefore, targeting N-cadherin may be a promising therapeutic approach, particularly in cisplatin-resistant, therapy refractory and metastatic GCT.

## INTRODUCTION

Germ cell tumors (GCTs) are the most common malignancies in young men between 15–40 years. The incidence of GCT has been constantly increasing over the last 40 years [1]. Distinction of the different histological subtypes is essential for the therapeutic management. Non-seminomas can be further subdivided into embryonic carcinomas (EC), yolk sac tumors (YS), chorionic carcinomas (CC), and teratomas (TER) [2]. Seminomas and non-seminomas have a common precursor called intratubular germ cell neoplasia (IGCNU) [3]. Patients with metastatic GCTs can be cured in about 80% of cases by using cisplatin-based combination chemotherapy [4, 5]. However, patients with multiple relapses have an unfavorable prognosis, and long-term survival can be achieved in only 10–15% of these patients [6, 7]. Therefore, new treatment options for refractory GCTs are needed.

For *in vitro* studies, several GCT cell lines are available. TCam-2 shows seminoma characteristics, whereas NCCIT and NTERA-2 model embryonic carcinomas [8, 9]. Two cisplatin-resistant GCT cell lines, NTERA-2R and NCCIT-R, were established to investigate mechanisms of cisplatin resistance *in vitro* [10].

Cadherins are Ca^2+^-dependent transmembrane glycoproteins belonging to the group of adhesion molecules. More than 80 different members of cadherins are known, such as the well-investigated epithelial, neural, and placental cadherins [11]. Cadherins play a crucial role in cell-cell contacts, during embryonic organ development, but also in the biology of several tumors. In addition, cadherins can act as metastasis-suppressing proteins [12, 13]. N-cadherin (CDH2) is a 140 kDa protein and was first identified in mouse brain tissue [14]. It plays an important role in migration, differentiation, embryonic development, and metastatic behavior of tumor cells [15]. N-cadherin associates with the actin-cytoskeleton through interactions with cytoplasmic catenin proteins [16,17]. N-cadherin expression was observed in neoplastic tissues of epithelial and mesenchymal origin such as tumors of the lung, ovary, and kidney, but also in different normal tissues [18–24]. We have previously shown that N-cadherin shows a differential expression pattern in the histological subtypes of GCTs [25].

In the present study, we used parental GCT cell lines TCam-2, NCCIT and NTERA-2 and their cisplatin-resistant sublines to further investigate the expression and functional role of N-cadherin and as a model of cisplatin resistance in GCT.

## RESULTS

### N-cadherin protein is expressed in cisplatin-sensitive and resistant GCT-cell lines

**Figure 1:**
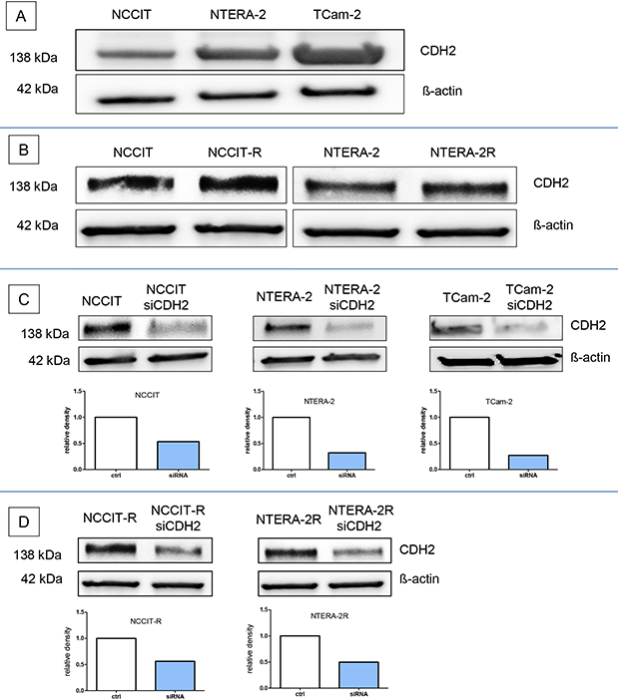
N-cadherin protein is expressed in cisplatin-sensitive and resistant GCT-cell lines N-cadherin protein expression was found in the GCT cell lines NCCIT, NTERA-2, and in TCam-2 cells **A.** and the two cisplatin-sensitive and –resistant cell line pairs NCCIT/-R and NTERA-2/-R **B.** The siRNA against CDH2 (siCDH2) efficiently reduced N-cadherin expression in all investigated GCT cell lines **C+D.**

In western blot analysis, N-cadherin protein expression was found in all GCT-cell lines examined, namely NCCIT, NTERA-2, and their cisplatin-resistant sublines, as well as in TCam-2 cells. The expression was considerably higher in TCam-2 cells than in NCCIT or NTERA-2 (Figure 1A). No difference in N-cadherin protein expression levels was detected between the two cisplatin-sensitive and –resistant cell line pairs NCCIT/-R and NTERA-2/-R (Figure 1B).

### N-cadherin silencing in GCT cell lines by siRNA

The siRNA against *CDH2* (siCDH2) efficiently reduced N-cadherin expression in all investigated GCT cell lines. The relative density of the western blot bands was considerably reduced (Figure 1C+1D).

### N-cadherin expression in mouse xenografts

**Figure 2:**
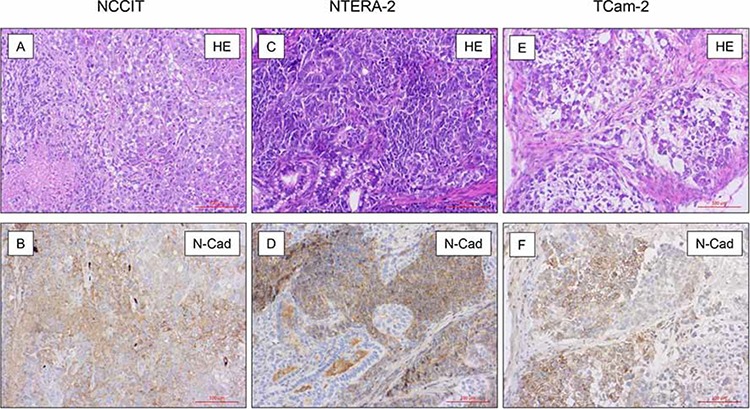
N-cadherin expression in mouse xenografts On immunohistochemical analysis in xenografts of NCCIT (*n* = 4; **A + B.**), NTERA-2 (*n* = 4; **C + D.**) and TCam-2 (*n* = 4; **E + F.**) N-cadherin was expressed in the cytoplasm and on the membrane of the tumor cells. The, expression of N-cadherin was higher in NTERA-2 and NCCIT, whereas the expression was lower in TCam-2 xenografts.

Xenografts of NCCIT (*n* = 4), NTERA-2 (*n* = 4) and TCam-2 (*n* = 4) were investigated for expression of N-cadherin protein. Formalin fixed and paraffin embedded tissues were investigated by immunohistochemistry as described above. N-cadherin was expressed in the cytoplasm and on the membrane of the tumor cells in NCCIT (Figure 2A+2B), NTERA-2 (Figure 2C+2D), and TCam-2-xenografts (Figure 2E+2F). Interestingly, in xenografts, expression of N-cadherin was higher in NTERA-2 and NCCIT, whereas the expression was lower in TCam-2 xenografts, therefore showing an opposite pattern to the expression results found by Western Blotting (see above).

### N-cadherin expression in metastasis of GCT

**Figure 3:**
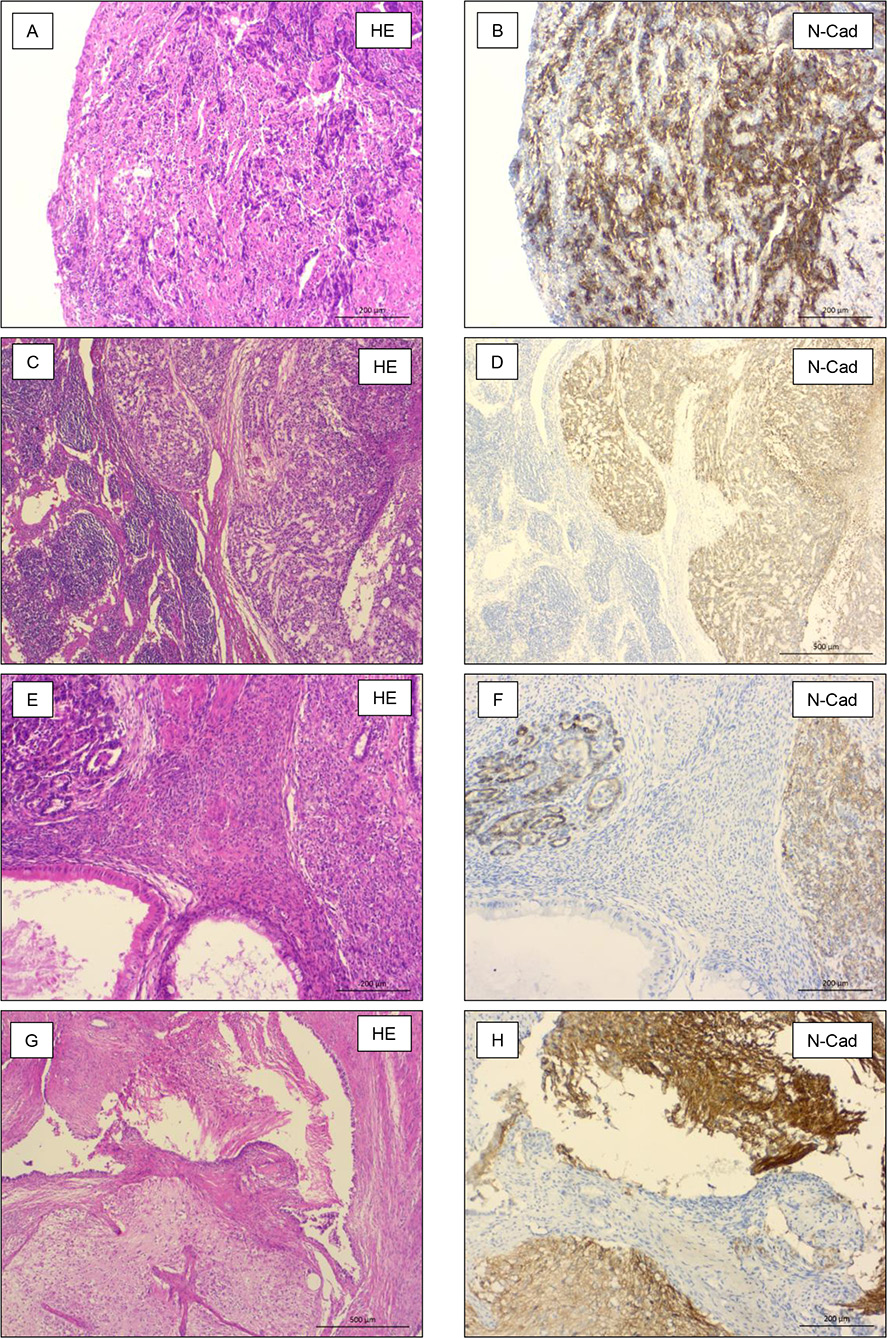
N-cadherin expression in metastasis of TGCT Seminomas (*n* = 3, **A + B.**) and yolk sack tumors (*n* = 5, **C + D.**) strongly expressed N-cadherin. Primitive neuroectodermal tissues (*n* = 4, **E + F.**) and neuronal tissue (*n* = 14, G+H) within mature teratomas strongly expressed N-cadherin, also.

Metastases of 28 patients with a primary testicular germ cell tumour were investigated for their expression of N-cadherin protein. Table 1 gives an overview of the different histological subtypes of investigated metastases. All metastases of seminomas (*n* = 3, Figure 3A+3B) and yolk sack tumours (*n* = 5, Figure 3C+3D) strongly expressed N-cadherin. In all investigated metastases of mature teratomas (*n* = 14), most areas were negative for N-cadherin. Some areas with intestinal epithelium and neuronal tissue show weak expression of N-cadherin. Neuroectodermal tissues within mature teratomas (*n* = 4) showed strong positivity for N-cadherin (Figure 3E–3H). Two metastases of embryonal carcinomas did not express N-cadherin (Figures not shown). An overview of investigated tumour types is listed in Table 1.

**Table 1:** overview of N-cadherin expression in metastases of primary GCT

Histological type of the metastases		N-cadherin staining
Seminoma	*n* = 3	3
Yolk sack tumor	*n* = 5	2
Mature teratoma	*n* = 14	1
Immature teratoma	*n* = 4	2
Embryonic carcinoma	*n* = 2	0

### Silencing of N-cadherin significantly reduces proliferation in GCT cell lines irrespective of cisplatin resistance

**Figure 4:**
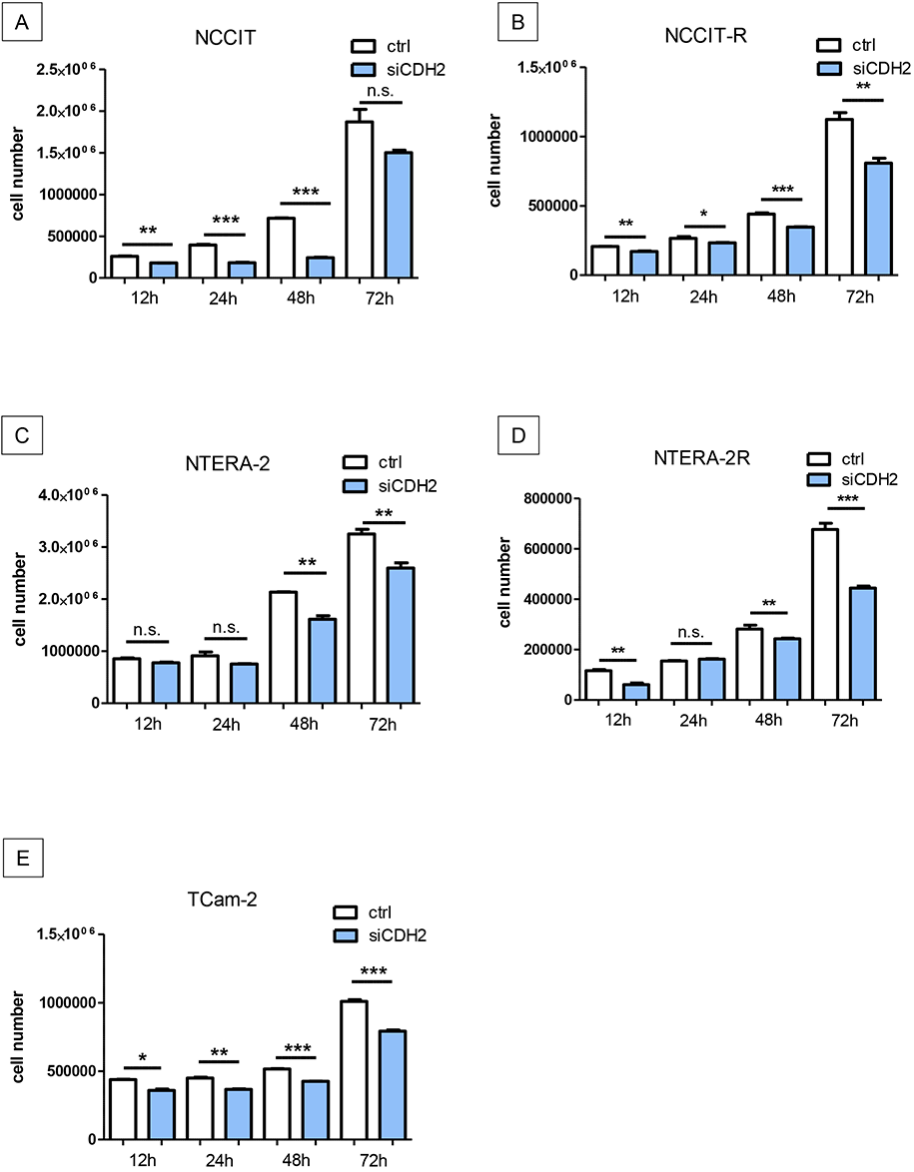
Silencing of N-cadherin significantly reduces proliferation in GCT cell lines Proliferation of NCCIT was significantly reduced after 12 h, 24 h and 48 h. After 72 h, no significant change in proliferation was distinguishable **A.** Proliferation of NCCIT-R was significantly reduced after 12 h, 24 h, 48 h and 72 h **B.** In NTERA-2, proliferation was significantly reduced after 48 h and 72 h. After 12 h and 24 h, no significant reduction of proliferation was detectable **C.** In NTERA-2R, proliferation was significantly reduced after 12 h, 48 h and 72 h. After 24 h, no significant reduction of proliferation was detectable **D.** The proliferation of TCam-2 was significantly reduced after 12 h, 24 h, 48 h and 72 h **E.** (n.s. = not significant, *= *p* < 0.05, **= *p* < 0.005, ***= *p* < 0.0005).

NCCIT/-R, NTERA-2/-R, and TCam-2 cell lines were transfected with siRNA against CDH2 for 12 h, 24 h, 48 h, or 72 h, as described above. Proliferation of NCCIT was significantly reduced after 12 h (*p* < 0.005), 24 h (*p* < 0.0005) and 48 h (*p* < 0.0005). After 72 h, no significant change in proliferation was observed (Figure 4A). Proliferation of NCCIT-R was significantly reduced after 12 h (*p* < 0.005), 24 h (*p* < 0.05), 48 h (*p* < 0.0005) and 72 h (*p* < 0.005) (Figure 4B). In NTERA-2, proliferation was significantly reduced after 48 h (*p* < 0.005) and 72 h (*p* < 0.005). After 12 h and 24 h (*p* < 0.005), no significant reduction of proliferation was detectable (Figure 4C). In NTERA-2R, proliferation was significantly reduced after 12 h (*p* < 0.005), 48 h (*p* < 0.005) and 72 h (*p* < 0.0005). After 24 h, no significant reduction of proliferation was detectable (Figure 4D). The proliferation of TCam-2 was significantly reduced after 12 h (*p* < 0.05), 24 h (*p* < 0.005), 48 h (*p* < 0.0005) and 72 h (*p* < 0.0005) (Figure 4E).

In addition, NCCIT/-R, NTERA-2/-R, and TCam-2 cell lines were blocked with an anti-N-cadherin antibody for 1 h, 12 h, 24 h and 48 h. In NCCIT, the proliferation was significantly reduced after 12 h (*p* < 0.0005), 24 h (*p* < 0.0005) and 48 h (*p* < 0.005). After 1 h, no significant reduction of proliferation was detectable (Supplementary Figure S1A). Proliferation of NCCIT-R was significantly reduced after 12 h (*p* < 0.05) and 48 h (*p* < 0.05). After 1 h and 24 h no significant reduction of proliferation was found (Supplementary Figure S1B). In NTERA-2, proliferation was significantly reduced after 1 h (*p* < 0.05), 12 h (*p* < 0.005), 24 h (*p* < 0.05) and 48 h (*p* < 0.05) (Supplementary Figure S1C). Proliferation of NTERA-2R was significantly reduced after 48 h (*p* < 0.05). After 1 h, 12 h 24 h no significant reduction of proliferation was detectable (Supplementary Figure S1D). Proliferation of TCam-2 was also significantly reduced after 1 h (*p* < 0.005), 12 h (*p* < 0.0005), 24 h (*p* < 0.0005) and 48 h (*p* < 0.0005) (Supplementary Figure S1E).

### Migration and invasion is significantly reduced after silencing of N-cadherin

**Figure 5:**
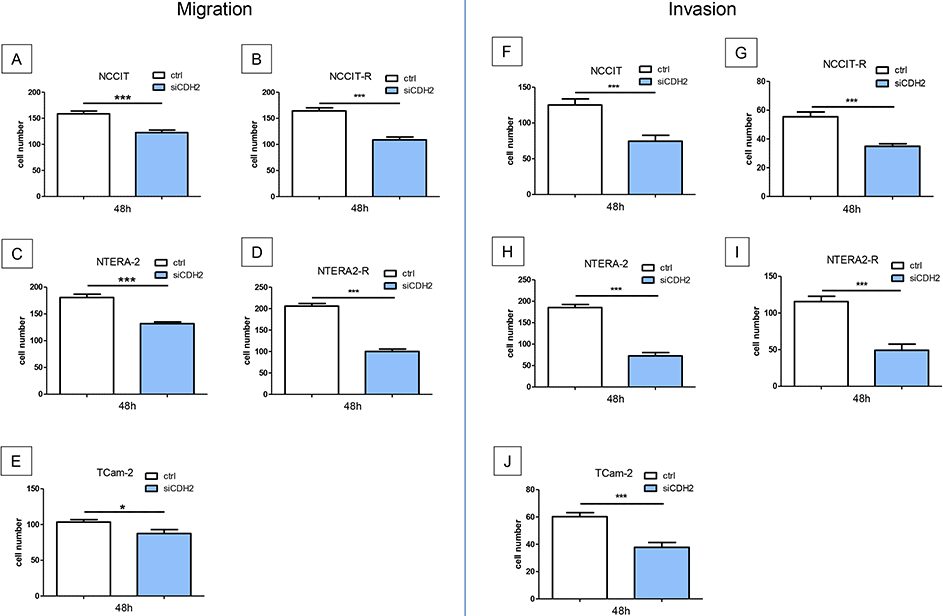
Migration and invasion is significantly reduced after treatment with a siRNA against CDH2 for 48 hours Migration of NCCIT **A.** NTERA-2 **B.** and TCam-2 **C.** NCCIT-R- **D.** and NTERA-2R- **E.** tumor cells was significantly reduced. Invasiveness of NCCIT **F.** NTERA-2 **G.** and TCam-2 **H.** NCCIT-R- **I.** and NTERA-2R- **J.** tumor cells was significantly reduced. (n.s. = not significant, *= *p* < 0.05, **= *p* < 0.005, ***= *p* < 0.0005).

Migration of NCCIT (*p* < 0.0005), NTERA-2 (*p* < 0.0005) and TCam-2 (*p* < 0.005) cells was significantly reduced after treatment with a siRNA against CDH2 for 48 hours. Likewise, migration of NCCIT-R- (*p* < 0.0005) and NTERA-2R- (*p* < 0.0005) cells was significantly reduced after treatment with a siRNA against CDH2 for 48 hours (Figure 5A–5E).

Invasiveness of GCT cell lines was investigated after siRNA transfection for 48 h. NCCIT (*p* < 0.005), NTERA-2 (*p* < 0.0005) and TCam-2 (*p* < 0.0005) tumor cells showed significantly reduced invasiveness. After treatment with a siRNA against CDH2 for 48 hours, the invasiveness of cisplatin resistant cell lines NCCIT-R- (*p* < 0.0005) and NTERA-2R- (*p* < 0.0005) cells was significantly reduced (Figure 5F–5J).

In addition, migration of NCCIT (*p* < 0.05), NTERA-2 (*p* < 0.005) and TCam-2 (*p* < 0.0005) cells was significantly reduced after blocking N-cadherin with a specific antibody for 24 hours (Supplementary Figure S2A–S2C). Invasiveness of NCCIT (*p* < 0.005), NTERA-2 (*p* < 0.005), and TCam-2 (*p* < 0.0005) cells could be significantly inhibited by the use of a specific antibody blocking N-cadherin for 24 hours (Supplementary Figure S2D–S2F).

### Downregulation of N-cadherin leads to significant higher levels of pERK

**Figure 6:**
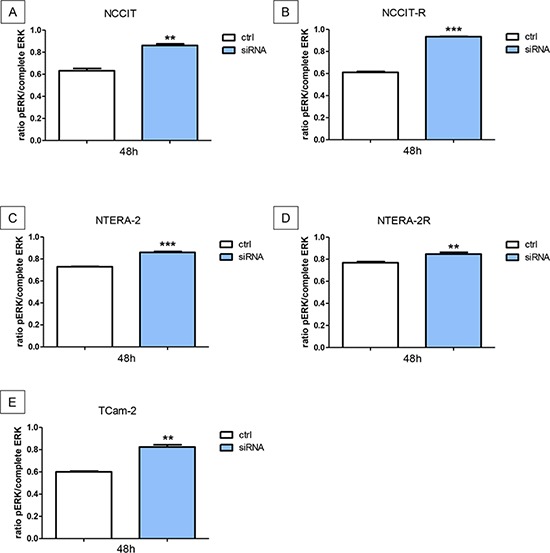
Downregulation of N-cadherin leads to significant higher levels of pERK The downregulation of N-cadherin leads to a significant increase of pERK in NCCIT **A.** NCCIT-R **B.** NTERA-2 **C.** NTERA-2R **D.** and TCam-2 **E.** cell lines (n.s. = not significant, *= *p* < 0.05, **= *p* < 0.005, ***= *p* < 0.0005).

Cisplatin-sensitive and resistant GCT cell lines were investigated for pERK. The downregulation of N-cadherin leads to a significant increase of pERK in NCCIT (*p* < 0.005), NCCIT-R (*p* < 0.0005), NTERA-2 (*p* < 0.0005), NTERA-2R (*p* < 0,005), and TCam-2 (*p* < 0,005) cells (Figure 6A–6E).

### Downregulation of N-cadherin has no influence on pAKT in cisplatin-sensitive and resistant GCT cell lines

Cisplatin-sensitive and resistant GCT cell lines were investigated for pAKT after transfection with siRNA against N-cadherin. In the cell lines NCCIT, NCCIT-R, and NTERA-2R, levels of pAKT showed no differences after siRNA transfection. In addition, pAKT was significantly decreased in NTERA-2 (*p* < 0.005) and TCam-2 (*p* < 0.05) cells after siRNA transfection (Supplementary Figure S3A–S3E).

### Blocking N-cadherin induces apoptosis

**Figure 7:**
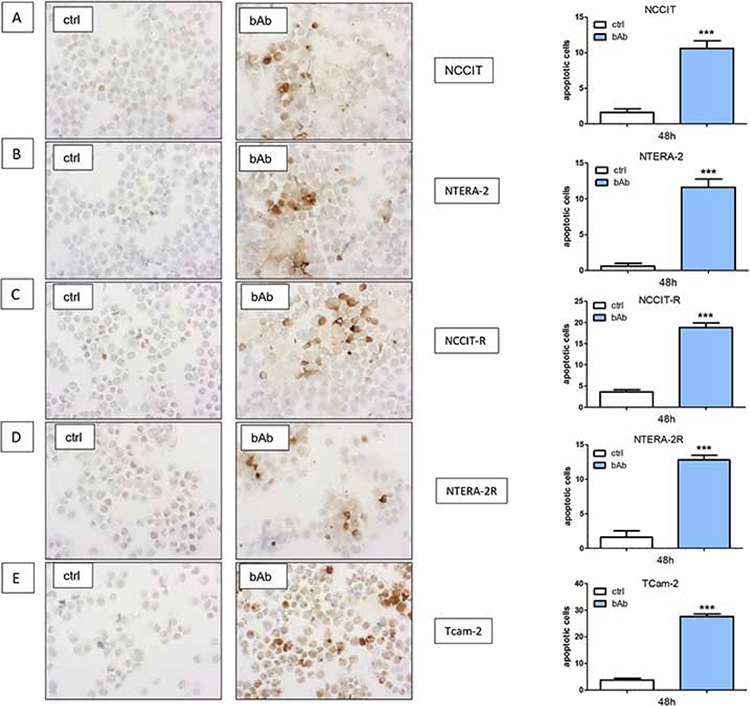
Blocking N-cadherin causes significantly higher numbers of apoptotic cells GCT cell lines NCCIT **A.** NCCIT-R **B.** NTERA-2 **C.** NTERA-2R **D.** and TCam-2 **E.** showed a significant increase in the number of apoptotic cells in immunocytochemistry analysis for Caspase-3. (n.s. = not significant, *= *p* < 0.05, **= *p* < 0.005, ***= *p* < 0.0005).

GCT cell lines were treated with a blocking antibody against N-cadherin. All cell lines showed a significant increase of apoptotic cells in immunocytochemistry analysis of Caspase-3 (NCCIT, NCCIT-R, NTERA-2, NTERA-2R and TCam-2; *p* < 0.0005; Figure 7A–7E).

## DISCUSSION

New treatment options for refractory TGCTs are desperately needed. Recently, the mechanisms of cisplatin resistance are beginning to emerge, and potential drug targets have been described [26]. Nevertheless, new therapeutic options and a better understanding of potential mechanisms of resistance are needed.

In a variety of normal tissues (e.g. neuronal, endothelial and muscle cells) expression of N-cadherin has been reported [27, 28]. Moreover, N-cadherin expression has been described in several tumor entities, including GCT. In the case of GCT, N-cadherin is expressed in normal testis, precursor lesions, seminomas, and yolk sack tumors. In contrast, no expression could be detected in embryonic carcinomas and chorionic carcinomas in formalin-fixed, paraffin-embedded material [25]. Interestingly, GCT cell lines, some of them of embryonal carcinoma origin, express N-cadherin in western blot analysis, as shown in this study. In addition, we used unfixated (fresh) tissue of embryonal carcinoma for analysis of N-cadherin protein expression. The results of western blot analysis demonstrate N-cadherin expression, albeit at varying levels, in embryonal carcinoma (Supplementary Figure S4). This difference in N-cadherin expression might be attributable to changes occurring during the fixation process. We used the two representative embryonic carcinoma cell lines NCCIT/-R and NTERA-2/-R as well as TCam-2 to investigate the role of N-cadherin in GCT.

N-cadherin is able to induce cell survival, migration, and invasion by modulating intracellular signaling molecules. Altered N-cadherin expression has been described during epithelial mesenchymal transformation (EMT) [29]. The stem cell genes Oct-4 und Nanog, among other, promote EMT in breast cancer stem cells. The co-knockdown of Oct-4 and Nanog significantly reduced the relative N-cadherin protein expression levels [30]. In GCT, reduced Oct-4 expression has been described in cisplatin resistance [31], and Gutekunst et al. could show that in GCTs Oct-4–dependent high constitutive Noxa levels are required for cisplatin hypersensitivity [32]. In contrast, we could not demonstrate differences in N-cadherin expression when comparing cisplatin-sensitive and resistant cell line pairs, suggesting that GCT might show differences in pathways involved in EMT, compared to other solid tumors. However, cross-talk of N-cadherin with other membrane proteins activates signaling cascades, thus influencing cell proliferation or invasion [28]. N-cadherin blockade has been shown to lead to an inhibition of cancer growth, metastasis formation, and delay castration resistance in prostate cancer. In this study, silencing of N-cadherin reduced activation of AKT [33]. As shown in our investigations, downregulation of N-cadherin reduces proliferation, migration and invasion in parental and cisplatin-resistant GCT sublines *in vitro*.

The N-cadherin signaling pathway seems to be variable. On the one hand, Li et al. described the role of N-cadherin in melanoma cells. In this study, a knockdown of N-cadherin with a blocking antibody leads to a significant reduction of cell survival. However, in melanoma cells, N-cadherin blocking inhibited Akt/PKB phosphorylation [31]. It has also been shown that N-Cadherin decreases osteoblast proliferation or survival by antagonizing Pi3K/AKT signaling [33]. S Chung et al. described the regulation of mammary tumor cell migration by AKT3 suppression through N-cadherin [32]. Our data suggest that blocking N-cadherin in GCT cell lines leads to a significantly higher level of p-ERK, whereas the activation of p-AKT does not seem to play an important role in cisplatin-sensitive and resistant GCT cells. Interestingly, an earlier analysis demonstrated by immunohistochemistry, that there was no clear pattern of Akt activation in non-seminomas [34]. Nalla et al. could demonstrate that in prostate cancer cells, the knockdown of N-cadherin increased ERK phosphorylation [35]. This would be in line with the data in our study. It could previously be shown that cisplatin induces apoptosis in GCT cell lines through an activation of the MEK–ERK signaling pathway. Schweyer et al. showed that the activation of ERK culminated in activation of the executor caspase-3 [36]. As shown in this study, the inhibition of N-cadherin leads to significantly reduced proliferation and higher levels of p-ERK. By immunocytochemical analysis of GCT cell lines, significantly increased levels of apoptosis in the caspase-3 staining could be detected following N-cadherin blockade. Our data suggest that the underlying mechanism by which the blockade of N-cadherin could reduce proliferation may depend on activation of p-ERK. Xenografts of GCT cell lines in nude mice showed N-cadherin expression in all samples analysed. Therefore, xenografts of these cell lines could be a valid cell model to investigate effects of N-cadherin blockade *in vivo*. In summary, downregulation or blocking of N-cadherin leads to a significant decrease of proliferation, migration, and invasion in GCT cell lines, irrespective of cisplatin resistance levels. The inhibition of N-cadherin leads to activation of p-ERK, and causes apoptosis. Thus, targeting N-cadherin may be a promising therapeutic approach, particularly in cisplatin-resistant GCT, where current options are limited. As shown in this study, metastases of primary GCT express N-cadherin. Interestingly, when comparing primary GCT and metastases, no differences concerning N-cadherin expression were detected. Since N-cadherin is expressed on the cell surface, it could be a potential therapeutic target, e.g. for therapeutic antibodies. A different approach is the use of small molecules like ADH-1 (Exherin^TM^), which is an N-cadherin antagonist cyclic pentapeptide. Clinical phase I studies could show that ADH-1 was generally well tolerated and showed evidence of anti-tumor activity in patients with N-cadherin positive tumors, among others in refractory solid tumors [37–39]. This may be a new therapeutic option, especially in N-cadherin positive GCT.

## MATERIALS AND METHODS

### Culture of GCT cell lines

In the present study, the human GCT-cell lines NCCIT (teratocarcinoma, CRL 2073), NTERA-2 (embryonic carcinoma, CRL 1973, both cell lines from American Type Culture Collection, Manassas, VA, USA) and TCam-2 (seminoma; kindly provided by H. Schorle, Department of Developmental Pathology, University of Bonn Medical School, Germany) were used. Both cisplatin-resistant sublines NCCIT-R and NTERA-2R (kindly provided by C. Jacobsen, University Hospital Hamburg-Eppendorf, Germany) were cultured as described previously [10]. Cell lines were cultured in HEPES-buffered RPMI-1640 (Biochrom, Berlin, Germany) supplemented with fetal calf serum (FCS, 10%; CC Pro, Neustadt, Germany), penicillin (100 IU/ml; Sigma, Munich, Germany), streptomycin (100 μg/ml; Sigma), and L-glutamine (2 mM; Biochrom). The incubation temperature was 37°C in a humid atmosphere with 5% carbon dioxide in the air.

### Western blot analysis

Total protein lysates were prepared using RIPA buffer with protease inhibitors (Roche, Mannheim, Germany) and were quantified using the Bio-Rad DC Protein Assay (Bio-Rad, Hercules, California, USA). For western blot analysis, primary antibodies against anti-N-cadherin (polyclonal rabbit, sigma-aldrich, Taufkirchen, Germany; dilution 1:1000, 4°C overnight) and anti-beta-actin (monoclonal mouse, sigma-aldrich, dilution 1:5000, room temperature 1 hour) were used. Primary antibodies were detected by using anti-mouse secondary antibodies (Dako, Hamburg, Germany, dilution 1:1000). Membranes were developed with the ECL system (Amersham Bioscience, Freiburg, Germany).

### Mouse tumor xenografts

Six-week-old male athymic nude (BALB/c-nu) mice were purchased from Charles River Germany (Sulzfeld, Germany). For acclimatization, mice were maintained for 2 weeks in standard cages with air filter hoods, with free access to food and water. Subsequently, all animals received an s.c. inoculation of 1 × 10^6^ exponentially growing NTera-2, NCCIT and TCam-2 cells, resuspended in 100 μL PBS mixed with 100 μL Matrigel (Becton Dickinson GmbH, Heidelberg, Germany) on the dorsal portion of the forelegs via a 26-gauge needle. Animals were sacrificed after 60 days and tumors were excised. Tumors were fixed in 10% buffered formalin and embedded in paraffin. All experiments were performed with approval by the local animal protection committee (reference No. 33.11.42502-04-058/07).

### Immunohistochemistry

Immunohistochemistry of N-cadherin was performed on 4-μm formalin-fixed and paraffin-embedded tissue as described previously [25]. Tumour tissues from metastases of GCT specimens were acquired from the University Medical Centre Göttingen and were classified and staged on the basis of the WHO classification [40]. Ethical approval for using human material in the present study was obtained from the ethics committee of the University Medical Centre Göttingen. Sections were stained using a Dako autostainer with the Dako EnVision™ FLEX+ detection system (Dako, Glostrup, Denmark). The system detects primary mouse and rabbit antibodies, and the reaction was visualized by EnVision™ FLEX DAB+ Chromogen. Using EnVision™ FLEX+ Mouse (LINKER) or EnVision™ FLEX+ Rabbit (LINKER) (Code K8019), signal amplification of primary antibodies can be achieved. Deparaffinization, rehydration, and heat-induced epitope-retrieval (HIER) was carried out in one step with the 3-in-1 procedure Buffer (Dako, Glostrup, Denmark, Target Retrieval Solution), pH 9 high ((10x)(3-in-1) Code S2375)) at 97°C using a PT Link, Pre-Treatment Module 6 (Dako). Tissue samples were analysed by light microscopy after 8 min counterstaining with Meyer's haematoxylin (Dako). Two pathologists evaluated all tissue sections using an immunoreactive staining score (IRS). The percentage of positively stained cells was first categorized using a 0–4 scoring system: Score 0 = 0% positive cells, score 1 = less than 10% positive cells, score 2 = 10–50% positive cells, score 3 = 51–80% positive cells and score 4 = > 80% positive cells. The intensity of staining was evaluated on a graded scale (0 = negative; 1 = weak; 2 = intermediate; 3 = strong). For the final IRS, the scores of intensity and staining were multiplied and the mean value was calculated.

### N-cadherin blocking antibody

Tumor cells were blocked with a monoclonal anti-N-cadherin antibody produced in mouse (sigma-aldrich). After 24 hours of incubation, the culture medium was exchanged for the antibody mix with a concentration of 10 μg/ml. In addition, 10 μl antibody (IgG concentration 2–2.5 mg/ml) was mixed with 2ml culture medium. The cells were incubated for 1 h, 12 h, 24 h, or 48 h.

### siRNA transfection

Tumor cells were transfected with 100 μl anti N-cadherin siRNA medium (12 μl Hiper feet, 2,5 μl siRNA and 85,5 μl RPMI medium). siRNA used was Hs_CDH2_6, cat. nr. SI02757335 (Quiagen, Hilden, Germany). After incubation for 20 min, 100 μl siRNA medium were mixed with 2.3ml culture medium to create a concentration of 1:1000. The cells were incubated for 12 h, 24 h, 48 h, or 72 h respectively. Si-RNA aggradation in the cells was measured by FACs-analysis with fluorescent siRNA in the same concentration as described above.

### Immunocytochemistry

Tumor cells were treated as described above and subsequently transfered onto slides using cytospin at 1000 rpm for 10 minutes. The slides were allowed to dry overnight. After fixation with formalin for 15 minutes, the slides were rinsed with PBS. To permeabilize the cells, we used PBS with 0.25% Triton X-100 for 5 minutes. The endogenous peroxidase was blocked with 3%H2O2 for 5 minutes. The Caspase3-Antibody was applied afterwards (cleaved Caspase 3, 1/50, Zytomed Systems RBK009–05) for 1 hour. The following secondary antibody (anti mouse/rabbit, labelled with HRP) was incubated for 25 minutes and the chromogen (DAB) for 5 minutes (Dako REAL EnVision Detection System, DAKO). Samples were counterstained with Meyer's Haematoxylin and covered.

### Assessment of cell proliferation

1 × 10^5^ to 3 × 10^5^ cells were plated as described above. After 24 h incubation time, the culture medium was exchanged for an antibody-medium (2 ml culture medium, 20 μl N-cadherin-antibody) or a siRNA-medium (2.3 ml culture medium, 100 μl siRNA-Mix). Cells were dissolved with trypsin. 50 μl cell suspension was supplemented with 450 μl proliferation kit. Cell proliferation was measured with flow cytometry.

### Migration assay

For *in vitro* cell migration, GCT cell lines were treated with N-cadherin-blocking antibody or siRNA for 24 or 48 h. Subsequently, 7 × 10^4^ cells were transferred into the Millicell 8.0 μm hanging PET inserts (Millipore, Billerica, MA) with 10% FCS. To create a gradient, the medium in the culture plates contained 20% FCS. The cells were incubated for 24 h as described above. Afterwards, cells were counterstained with Meyer's Haematoxylin (Dako) for 2 min, and analyzed by light microscopy.

### Invasion assay

The *in vitro* cell invasion from TGCT cells was determined using BioCoat Matrigel Invasion Chambers (BD Pharmingen™, San Diego, California, USA). Cells were pre-treated with N-cadherin-blocking antibody or siRNA for 24 or 48 hours. 70 000 cells were plated in medium containing 10% FCS into the inserts. The medium in the culture plates contained 20% FCS to create a gradient. The cells were incubated for 48 h, fixed, and counted microscopically.

### MAPK activation dual detection kit

The Dual Detection kits each contain two directly conjugated antibodies, a phospho specific antibody (PI3K: phospho-specific anti-phospho-Akt (pAKT, Ser473) Alexa Flour555; MAPK: phosphor-specific anti-phospho-ERK1/2 (pERK, Thr202/Tyr204, Thr185/typ187)-Phycoerythrin) and a second antibody to measure the total levels of either ERK or Akt (PI3K: anti-Akt, PECy5 conjugated; MAPK: anti-ERK1/2-PECy5 conjugated). Each kit allows measuring of the phosphorylated level against the total amount of expression. Cells were plated in a 6-Well Plate with 300,000 Cells per well and simultaneously transfected with siRNA against N-Cadherin (as described above). After 48 h of treatment the cells were trypsinized and the cell number was determined using the MUSE Cell Count&Viability Kit (Merck Millipore). The volumes used in the cell preparation were dependent of the measured cell number. Cells were prepared and treated according to the MUSE Dual Detection Kit Protocol as described by the manufacturer. Cells were fixed on ice for 5 minutes after adding 500 μl Assay Buffer per one million cells and 500 μl Fixation Solution. After a washing step the permebilization is carried out on ice for 5 minutes as well by resuspending cells in 1ml cold permeabilization buffer per one million cells. Cells were washed again with assay buffer and resuspended in 450 μl assay buffer per one million cells and aliquoted à 90 μl. For staining with both antibodies, 10μ of a previously prepared antibody-cocktail is added to the cell suspension. After 30 minutes incubation in the dark, followed by a washing step with assay buffer to remove the remaining antibodies, the stained cells are resuspended in 200 μl assay buffer. To adjust the settings exactly for every cell line, a sample stained with 5 μl of only the one antibody to measure the total amount of the desired protein is prepared. The settings for each sample are adjusted with its own total expression. By doing so, the sample stained with both antibodies will show precisely the ratio between the total and the phosphorylated amount.

### Assessment of apoptotic cells

The cultured and treated cells (as described above) were spun down onto slides at 1000rpm for 10 minutes. The slides were allowed to dry overnight. After fixation with formalin for 15 minutes, the slides were rinsed with PBS. The cells were permeabilized using PBS with 0,25% Triton X-100 for 5 minutes. Endogenous peroxidase was blocked with 3%H2O2 for 5 minutes. Caspase3-Antibody was applied afterwards (cleaved Caspase 3, 1/50, Zytomed Systems RBK009-05) for 1 hour. The secondary antibody (anti mouse/rabbit, labelled with HRP) was incubated for 25 minutes and the chromogen (DAB) for 5 minutes (Dako REAL EnVision Detection System, DAKO). Samples were counterstained with Meyer's Haematoxylin and coverslipped.

## SUPPLEMENTARY FIGURES


