# Supplementary material for: Role of N-cadherin in proliferation, migration, and invasion of germ cell tumours
Source: Oncotarget. 2015 Oct 2;6(32):33426–37. doi: 10.18632/oncotarget.5288 (PMC4741776; doi:10.18632/oncotarget.5288)
Supplement: Supplementary file 1 [file oncotarget-06-33426-s001.pdf]

SUPPLEMENTARY FIGURES

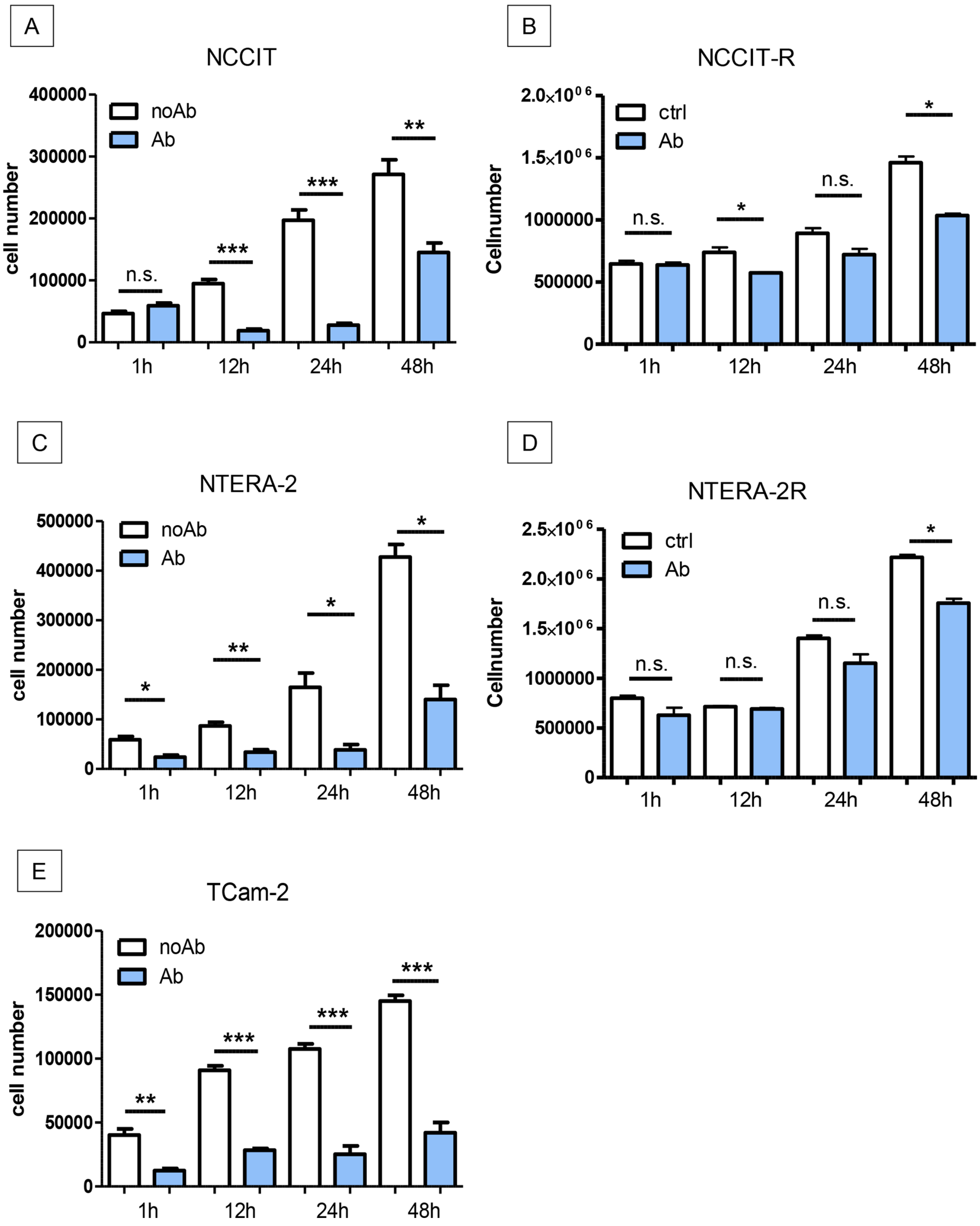

Supplementary Figure S1: N-Cadherin blockade leads to significant decrease of proliferation. In NCCIT cell line, the proliferation was significantly reduced after 12 h, 24 h and 48 h. After 1 h, no significant reduction of proliferation was distinguishable **A.** Proliferation of NCCIT-R was significantly reduced after 12 h, and 48 h. After 1 h and 24 h, no significant change in proliferation was distinguishable **B.** In NTERA-2, proliferation was significantly reduced after 1 h, 12 h, 24 h and 48 h **C.** In NTERA-2R, proliferation was significantly reduced after 1 h, 12 h, 24 h and 48 h **D.** Proliferation of TCam-2 was also significantly reduced after 1 h, 12 h, 24 h and 48 h **E.** (n.s. = not significant, *= $p < 0.05$, **= $p < 0.005$, ***= $p < 0.0005$).

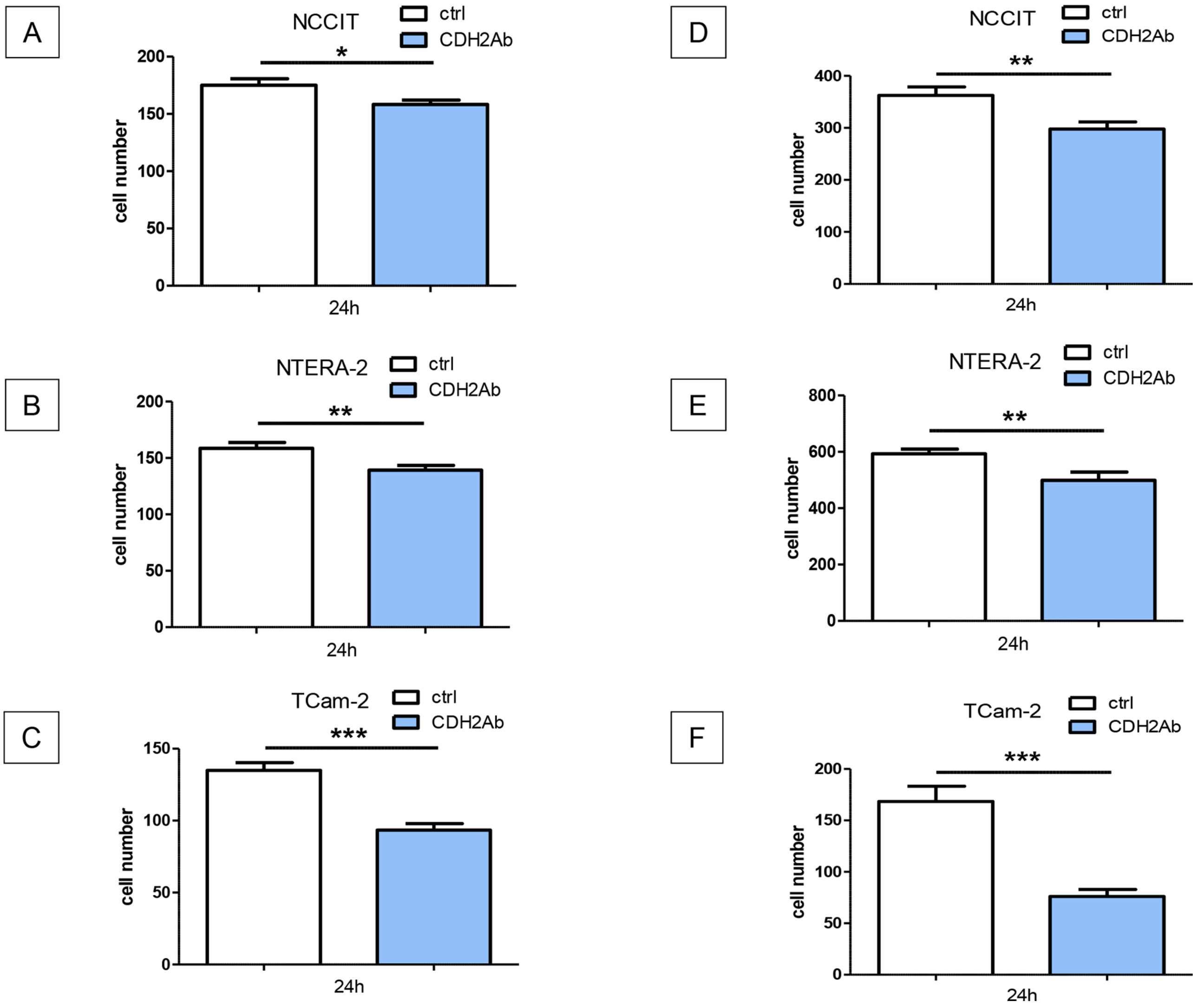

Supplementary Figure S2: Blockade of N-cadherin leads to significant decrease of migration and invasion. Migration of NCCIT **A.** NTERA-2 **B.** and TCam-2 **C.** cells was significantly reduced after blocking N-cadherin with a specific antibody for 24 hours. Invasiveness of NCCIT (C), NTERA-2 **D.** and TCam-2 **E.** cells could be significantly inhibited by the use of a specific antibody blocking N-cadherin for 24 hours. (n.s. = not significant, *= $p < 0.05$, **= $p < 0.005$, ***= $p < 0.0005$).

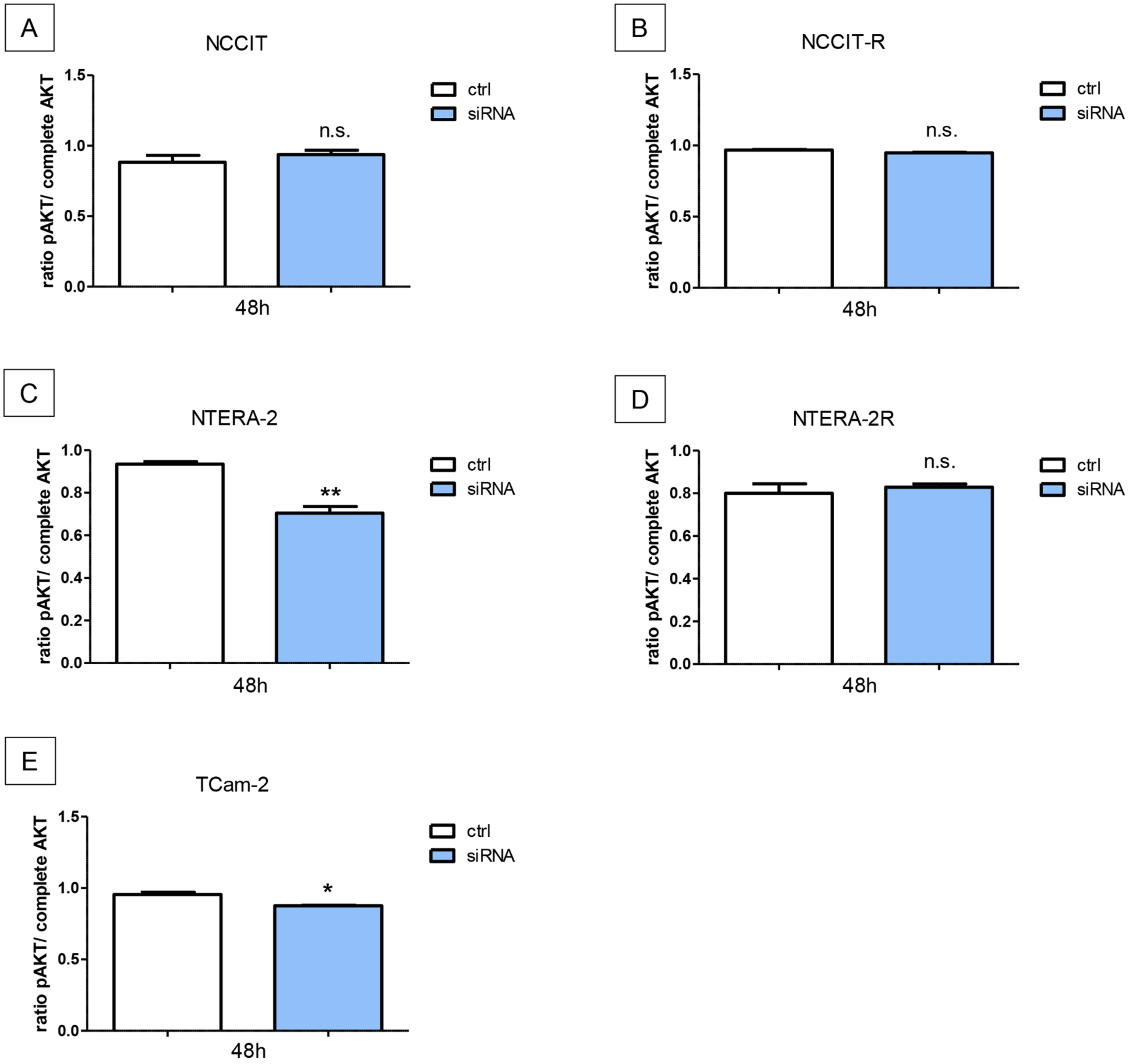

Supplementary Figure S3: Downregulation of N-cadherin has no effect on pAKT in cisplatin-sensitive and resistant GCT cell lines. Downregulation of N-cadherin had no effect on pAKT in NCCIT **A.** NCCIT-R **B.** NTERA-2R **D.** and TCam-2 **E.** In NTERA-2 levels of activated Pi3K were significantly increased in **C.** (n.s. = not significant, *= $p < 0.05$, **= $p < 0.005$, ***= $p < 0.0005$).

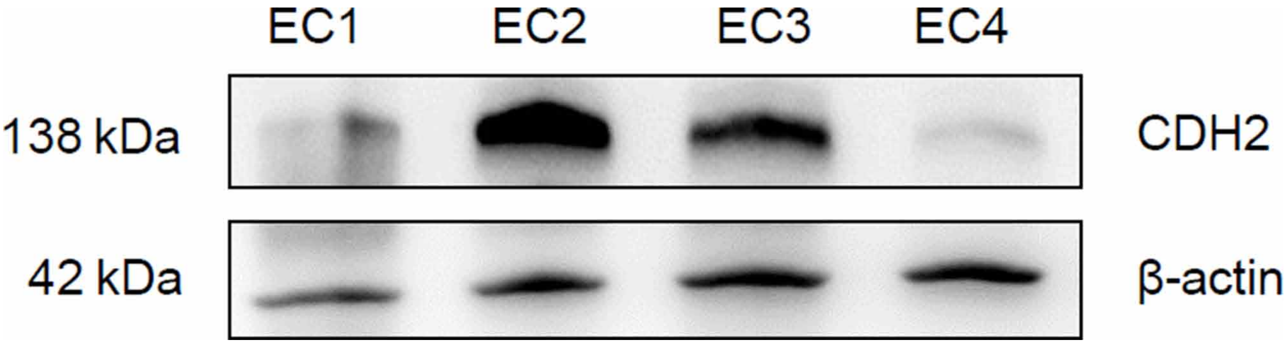

Supplementary Figure S4: N-cadherin expression in unfixated embryonal carcinoma tissue. Four embryonal carcinomas were analyzed for their N-Cadherin expression. While all tumors express N-cadherin in western blot analysis, the expression varies considerably between the different cases.
